# Warfarin Toxicity and Individual Variability—Clinical Case

**DOI:** 10.3390/toxins2112584

**Published:** 2010-10-28

**Authors:** Irina Piatkov, Colin Rochester, Trudi Jones, Steven Boyages

**Affiliations:** Diversity Health Institute, DHI Laboratory, ICPMR level 2, Sydney-West Area Health Service, Westmead Hospital, Westmead, NSW 2145, Australia; Email: colin.rochester@swahs.health.nsw.gov.au (C.R.); trudi.jones@swahs.health.nsw.gov.au (T.J.); steven.boyages@swahs.health.nsw.gov.au (S.B.)

**Keywords:** warfarin, pesticides, anticoagulant

## Abstract

Warfarin is a widely used anticoagulant in the treatment and prevention of thrombosis, in the treatment for chronic atrial fibrillation, mechanical valves, pulmonary embolism, and dilated cardiomyopathy. It is tasteless and colorless, was used as a poison, and is still marketed as a pesticide against rats and mice. Several long-acting warfarin derivatives—superwarfarin anticoagulants—such as brodifacoum, diphenadione, chlorophacinone, bromadiolone, are used as pesticides and can produce profound and prolonged anticoagulation. Several factors increase the risk of warfarin toxicity. However, polymorphisms in cytochrome P450 genes and drug interactions account for most of the risk for toxicity complications. Each person is unique in their degree of susceptibility to toxic agents. The toxicity interpretation and the health risk of most toxic substances are a subject of uncertainty. Genetically determined low metabolic capacity in an individual can dramatically alter the toxin and metabolite levels from those normally expected, which is crucial for drugs with a narrow therapeutic index, like warfarin. Personalized approaches in interpretation have the potential to remove some of the scientific uncertainties in toxicity cases.

## 1. Introduction

Each person is potentially unique in his or her susceptibility to the spectrum of possible toxic agents. Not surprisingly, there can be uncertainty in toxicity interpretation and the health risk of most toxic substances. Genetic data and new genetic methods have the potential to remove some of the scientific uncertainties in toxicity cases. 

The case report described in our article is an example intended to maintain awareness of the benefits of pharmacogenetic testing in conjunction with warfarin treatment and antidepressant medication. Despite recommendations from such bodies as the United States Food and Drug Administration (FDA), the practice has not been widely adopted. 

The patient presented with deep vein thrombosis and experienced ongoing bleeding during treatment with a standard warfarin dose according to International Normalized Ratio (INR) results. In the described case, instead of being a primary part of the treatment plan, genetic tests were performed only when no other cause could be found for these bleeding episodes. The tests helped in interpretation of the development of warfarin toxicity.

### 1.1. Warfarin Toxicity

Warfarin is a widely used anticoagulant in the treatment and prevention of thrombosis. It was initially marketed as a pesticide against rats and mice and is still used for this purpose. It was approved for use as a medication in the early 1950s and is widely prescribed. Despite its common use, warfarin therapy can be associated with significant bleeding complications. Achieving a safe therapeutic response can be difficult because of warfarin’s narrow therapeutic index and great individual variability in the dose required, which is mostly a consequence of individual genetic variants. This fact is well known among clinicians and a wide range, from 1 mg/day to 20 mg/day, of warfarin maintenance doses are observed across the population. To maintain a therapeutic level of anti-thrombosis and to minimize the risk of bleeding complications, warfarin therapy requires intensive monitoring via the INR to guide its dosing. The INR is used to monitor the effectiveness of warfarin and measures of the pathway of blood coagulation. The INR is used to standardize the results for a prothrombin time. The INR is the ratio of a patient’s prothrombin time to a control sample, raised to the power of the index value for the analytical system used.

Several factors increase the risk of over-anticoagulation: genetic polymorphisms affecting the metabolizing enzymes, impaired liver function, drug interactions, congestive heart failure, diarrhea, fever, and diets rich in vitamin K [1]. Nevertheless, genetic factors and drug interactions mostly account for the risk of over-anticoagulation. Warfarin metabolism involves primarily the cytochrome P450 (CYP) enzymes. Some loss-of-function CYP2C9 and VKORC1 polymorphisms are known to be associated with decreased enzymatic activity and as a result, with increased risk of hemorrhage. These are CYP2C9*2 (Cysl44/Ile359), CYP2C9*3 (Argl44/Leu359) [2] and VKORC1 (−1639G > A) [3].

Warfarin-induced hemorrhage is an important complication of anticoagulation therapy. A review of many studies shows average yearly rates of warfarin-related bleeding as high as 0.8%, 4.9%, and 15%, for fatal, major and minor bleeding complications, respectively [4].

Vitamin K is required by proteins C and S, together with clotting factors II, VII, IX, and X, to allow assembly of the procoagulant enzyme complexes necessary to generate fibrin. Warfarin as an anticoagulant agent has the ability to interfere with the recycling of vitamin K in the liver. The pharmacologic effect of warfarin is mediated by the inhibition of vitamin K epoxide reductase complex subunit 1 (EC 1.1.4.1) [5,6]. 

### 1.2. Warfarin Metabolism

**Figure 1:**
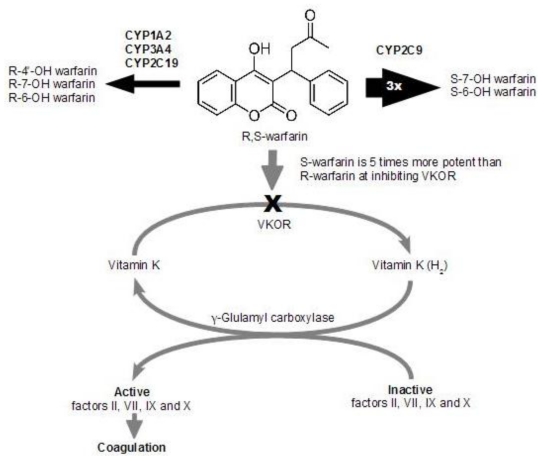
Warfarin metabolism. (Warfarin is metabolized in the liver. CYP1A1, CYP1A2, and CYP3A4 metabolize the (R)-enantiomer and CYP2C9 metabolizes the more potent (S)-enantiomer. Warfarin inhibits vitamin K reductase complex subunit 1 to interfere with the vitamin-K-dependent carboxylation of clotting factors prothrombin II, VII, IX, and X.).

Warfarin consist of (R)- and (S)-warfarin enantiomers. (R)- and (S)-warfarins differ in their relative plasma concentrations, in their antithrombotic potency and in the specific isoenzymes responsible for their metabolism. (S)-warfarin has a three-to-five times greater anticoagulant effect than the (R)-enantiomer and accounts for 60% to 70% of warfarin’s overall anticoagulant activity. (S)-warfarin is metabolized almost exclusively by CYP2C9 [7,8,9], see Figure 1.

The activity of the CYP2C9 enzyme has a significant impact on the clearance of (S)-warfarin and as a consequences on anticoagulant effect. In the presence of genetic variations where the activity of CYP2C9 is reduced, clearance of (S)-warfarin is also reduced. Activity of CYP2C9 between individuals can vary by more than 20-fold. (R)-warfarin is metabolized by multiple different CYP enzymes [10].

While several single-nucleotide polymorphisms of CYP2C9 have been reported, the CYP2C9*2 (Cysl44/Ile359) and CYP2C9*3 (Argl44/Leu359) polymorphisms have been identified as clinically relevant [11]. Both of these variants are associated with decreased enzymatic activity [12,13,14,15,16,17,18].

Homozygous CYP2C9*3 variant genotypes have only 5% to 10% metabolic efficiency compared to the wild-type genotype. As a result, compared to wild-type CYP2C9*1*1 controls, enzyme activity and the median maintenance warfarin dose for CYP2C9*3*1 heterozygotes was reduced by 40%, and by approximately 90% for CYP2C9*3*3 homozygotes [11,12,13].

Furuya [19] and Steward [14] showed that the CYP2C9*2 variant is also associated with reduced warfarin elimination. Heterozygotes demonstrate 40% and homozygotes 15% of the wild-type enzyme activity, causing dose adjustment for heterozygote CYP2C9*2 individuals down to 20% less than the standard dose.

Margaglione [15] has also demonstrated bleeding rates as high as 27.9 per 100 patient-years in carriers of CYP variants. In this study, findings were adjusted for other common variables associated with increased bleeding risk, such as increased age, drug interactions and abnormal liver function. 

Several studies of the *2 and *3 CYP2C9 polymorphisms consistently show that patients with at least one CYP2C9 allele polymorphism have reduced warfarin requirements [15,20,21,22,23,24]. 

Freeman [25] reported reduced warfarin weekly dosages for carriers of CYP2C9*2 or CYP2C9*3 alleles compared with patients who were homozygous for the wild-type allele (0.307 mg/kg/wk and 0.397 mg/kg/wk, respectively). 

Taube [23] compared warfarin maintenance dosages in 683 patients carrying different CYP2C9 genotypes. Mean warfarin maintenance dosages were 86% in patients with CYP2C9*1/CYP2C9*2, 79% in patients with CYP2C9*1/CYP2C9*3, 82% in compound heterozygotes CYP2C9*2/CYP2C9*3, and 61% in patients homozygous for CYP2C9*2. 

Furthermore, Aithal [20] warns that even when warfarin dosages are decreased, carriers of CYP2C9 poor metabolizer alleles experience a rate of major bleeding that is 3.68-fold higher than the rate seen in patients with the wild type genotype.

The frequency of CYP2C9 alleles is ethnically related [22,26]. Approximately 20% of the white population carries one of the loss-of-function CYP2C9 alleles, and it is estimated that 1% of whites carry two such alleles [10]. The frequency of the CYP2C9*2 allele reportedly ranges from 8–13% in different Caucasian populations. CYP2C9*2 is present in 4% of African-Americans and rare among Japanese individuals [27,28]. The frequency of CYP2C9*3 is 6–10% among Caucasian populations and 3.8% in Japanese populations [28,29]. This data suggests that a substantial fraction of the Caucasian patient population may carry at least one defective CYP2C9 allele. In this group, the usual prescription dosage of warfarin may lead to major or even life-threatening hemorrhage. 

### 1.3. Drug-drug Interaction

Drug-drug interaction is a main concern in adverse drug reactions. It has been shown that metronidazole and cimetadine increase the prothrombin time in patients on warfarin therapy. Chloramphenicol enhances warfarin’s effect by inhibiting the action of the hepatic P450 system [10].

Where depression coexists with cardiovascular disease, warfarin is commonly prescribed in combination with selective serotonin reuptake inhibitors (SSRIs). Case reports suggest that some SSRIs can interact with warfarin to increase the likelihood of bleeding [30]. SSRIs cause adverse effects in isolation [31,32] and can interact with other medications by inhibiting various isoenzymes of the CYP450 enzyme group [33,34].

The primary complication occurring with warfarin treatment is bleeding. SSRIs may increase the risk of bleeding during warfarin therapy by hindering platelet aggregation through depletion of platelet serotonin levels [35,36,37]. Some SSRIs may also inhibit the oxidative metabolism of warfarin by CYP2C9 [38]. 

Duncan [38] and Sayal [39] have warned that antidepressants with a known or predictable interaction with warfarin, such as fluoxetine and fluvoxamine, should be avoided in patients receiving warfarin because of the risk of adverse outcomes. 

## 2. Patient Case

We described a case of deep vein thrombosis and prolonged bleeding during treatment with a standard warfarin dose. The patient was a fifty year old woman on a treatment regime which included sertraline, flecainide, frusemide, potassium chloride, calcium and oestriol. 

The patient’s sample was referred to the Diversity Health Institute research laboratory when genetic hyperresponsiveness to warfarin was suspected. In the laboratory, genetic polymorphism analysis of the CYP2C9 gene was performed and the patient was found to be heterozygous for CYP2C9*2 and CYP2C9*3. 

The patient’s genotype—combined CYP2C9*2 and *3 polymorphisms—contributed to an increased anticoagulant response during the initiation of warfarin therapy at standard dose and later bleeding episodes through a reduced rate of drug breakdown. 

Furthermore, taking in consideration polydrug treatment, CYP2C19 and CYP2D6 genetic polymorphism were assessed. Analysis revealed that the patient also carries the loss-of-function polymorphisms CYP2D6*4 and CYP2D6*10, which are also associated with a poor metabolizer phenotype. CYP2D6 is responsible for the metabolism of endogenous and exogenous compounds. Sertraline as an inhibitor of CYP2D6 would possibly contribute to drug-interactions and consequent complications. In addition, as has been shown by Apsellof *et al.*, sertraline can increase free warfarin in the blood [40].

## 3. Discussion

As a heterozygote for CYP2C9*2 and CYP2C9*3, the patient experienced a typically exaggerated anticoagulant response during the initiation of warfarin therapy. As suggested in many publications described above, for patients who are known carriers of the CYP2C9 variant alleles, the initial loading doses of warfarin should be reduced by as much as 90% compared with the standard recommendation.

Coadministration of the antidepressant sertraline possibly contributed to the risk of abnormal patient bleeding, as it has been shown that concurrent use of selective serotonin reuptake inhibitors and warfarin increases the risk of hospitalization due to hemorrhage [30,36]. Drugs which affect serotonin may have a detrimental effect on platelet function, as drugs which inhibit the reuptake of serotonin may decrease platelet serotonin levels leading to a reduction in serotonin-mediated platelet aggregation. Potential drug interactions can involve modification in either of these mechanisms and may result in pharmacodynamic interference or enhancement of warfarin’s action.

Genotype analysis also revealed that the patient has the loss-of-function CYP2D6*4 and CYP2D6*10 polymorphisms, which are associated with the poor metabolizer phenotype. While there is no evidence of a CYP2D6 role in warfarin metabolism, this enzyme metabolizes many exogenous and endogenous substances. Taking into consideration that sertraline is a known inhibitor of CYP2D6 and that the patient is on a polydrugs treatment regime, this fact is probably contributing to the exacerbation of the patient’s complications. 

Knowledge of the individual genotype may influence the clinician’s decision to use warfarin, particularly in the high-risk or polydrug patients, or in patients with multiple genetic variations of metabolizing enzymes. Other anticoagulant agents, whose metabolism is not influenced by the CYP2C9 gene, are possibly a better choice in such circumstances.

Genetic testing has several advantages:

- Most laboratories do not perform blood levels of warfarin and its metabolites.
- There are uncertainties in the interpretation of the toxicity level.
- Daily repeated measurement of the prothrombin time (PT) and the international normalized ratio (INR) are the best to describe the anticoagulant effect. However, these tests may not be elevated until 1–2 days postingestion.
- Depressed levels of vitamin K-dependent clotting factors (II, VII, IX, and X) may provide supporting evidence for suspected warfarin toxicity, but these tests are rarely available in a timely fashion and usually do not aid in clinical management.
- Other laboratory tests, such as a blood count for baseline hemoglobin and/or hematocrit or to assess for anemia, only register changes when severe warfarin toxicity has occurred.
- Genotype assessment of the cytochrome P450 variant alleles may help the clinician to individualize treatment and minimize bleeding complications in patients who are poor metabolizers and need lower warfarin doses or complete drug substitution.

## 4. Conclusions

Accumulated scientific data suggests a correlation between individual genotype status and toxicity complications. Genotyping assays could provide valuable information that could be used in medical monitoring and toxicity interpretation. The question of whether a high drug metabolic ratio is due to poor enzyme capacity could be easily solved if the responsible gene is analyzed. The method could successfully be used for EDTA, heparin, fluoride or frozen blood samples, and even for material which was moderately decomposed or has been in long-term storage.

Evaluation of toxicological results includes several factors. Nevertheless, interactions between substances metabolized through the Phase I Cytochrome P450 system and an individual’s variation in the activity of enzymes participating in toxin metabolism obviously should be considered. 
